# Availability and Purchasing of Gluten-Free Cereal Products in a Polish Population of Female Celiac Disease Patients

**DOI:** 10.3390/foods14091495

**Published:** 2025-04-25

**Authors:** Dominika Guzek, Dominika Skolmowska, Dominika Głąbska, Frank Vriesekoop

**Affiliations:** 1 Department of Food Market and Consumer Research, Institute of Human Nutrition Sciences, Warsaw University of Life Sciences (SGGW-WULS), 159C Nowoursynowska Street, 02-776 Warsaw, Poland; dominika_guzek@sggw.edu.pl; 2 Department of Dietetics, Institute of Human Nutrition Sciences, Warsaw University of Life Sciences (SGGW-WULS), 159C Nowoursynowska Street, 02-776 Warsaw, Poland; dominika_skolmowska@sggw.edu.pl; 3 Harper Food Innovation, Harper Adams University, Newport TF10 8NB, UK; fvriesekoop@harper-adams.ac.uk

**Keywords:** celiac disease, gluten-free products, cereals, cereal products, bread, consumers, availability

## Abstract

Background/Objectives: The problems with following a gluten-free (GF) diet result from the high cost of GF products, their limited availability for celiac disease (CD) patients, and their disputable quality. The aim of this cross-sectional study was to assess the frequency of buying and availability of GF cereal products in a population of Polish female CD patients. Methods: This study was conducted in a population of Polish female CD patients who were members of the Polish Celiac Society, and *n* = 547 respondents were included in this study. Participants were asked about the frequency of buying and problems with the availability of GF cereal products, which were compared by sub-groups stratified by age, place of residence, place of purchasing major grocery shopping and purchasing GF products online. Results: The majority of the studied female CD patients declared often purchasing GF flour, pasta, and bread, as well as never purchasing GF puff pastry, fried baked goods, dumplings, and crackers. The only product for which the majority of the studied participants declared problems with availability was dumplings. For younger respondents, a higher share declared often buying GF pasta (*p* = 0.0073), chips, crisps and puffs (*p* < 0.0001), and Asian-style noodles (*p* = 0.0269), as well as declared problems with the availability of GF wraps/tortillas (*p* = 0.0001), puff pastry (*p* = 0.0294), fried baked goods (*p* = 0.0008), biscuits/cookies (*p* = 0.0148), and Asian-style noodles (*p* = 0.0046) compared to older respondents, while for older respondents, a higher share declared often buying GF flour (*p* = 0.0358), and never buying GF wraps/tortillas (*p* = 0.0181). For respondents living in big cities, a higher share declared problems with the availability of GF pasta compared to respondents living in small towns/villages (*p* = 0.0245). For respondents purchasing major grocery shopping in hypermarkets, a higher share declared often buying GF biscuits/cookies compared to respondents purchasing in other shops (*p* = 0.0039), while for respondents purchasing in other shops, a higher share declared never buying puff pastry (*p* = 0.0076), dumplings (*p* = 0.0002), and wraps/tortillas (*p* = 0.0038), as well as declared problems with availability of GF puff pastry (*p* = 0.0246), biscuits/cookies (*p* = 0.0002), and breakfast cereals (*p* = 0.0011). For respondents not purchasing GF products online, a higher share declared never buying GF fried baked goods compared to respondents purchasing online at least occasionally (*p* = 0.0284), as well as a lower share declared problems with the availability of GF wraps/tortillas (45% vs. 33%, *p* = 0.0411). Conclusions: The population of Polish female CD patients seems quite diverse in terms of the chosen GF cereal products, with age, primary place of purchasing major grocery shopping and purchasing GF products online, but not the place of residence, as the major determinants. The declared problems with the availability of GF products are probably associated with two diverse mechanisms—either frequent purchasing (as individuals not purchasing may not be interested in such a product at all) or rare purchasing (which may result from poor availability). Increasing the availability of GF cereal products for a population of Polish female CD patients may allow them to obtain a more diverse diet.

## 1. Introduction

Celiac disease (CD) is a chronic autoimmune disease associated with inappropriate immune-mediated reaction to consumed gluten, leading to a broad spectrum of gastrointestinal symptoms (resulting from damage to enterocytes) and extraintestinal symptoms (as a consequence of chronic inflammation and resultant nutrient malabsorption) [1]. Based on the systematic review and meta-analysis by King et al. [2], it was stated that the CD incidence in developed countries has been increasing in the second half of the 20th century and in the 21st century, while a higher incidence is observed for female compared to male individuals.

Gluten is a protein complex consisting of gliadin and glutenin, naturally found in certain cereal grains (e.g., wheat, barley, rye), and the cereal products made of them (e.g., bread, flour, pasta, noodles, groats, cereals, pastries, cakes) [3]. However, the other possible sources of gluten are non-certified products made of other cereals, as they may be processed on the same production line as gluten-containing grains, which may result in gluten contamination [4]. Another problematic issue for celiac patients is that other non-cereal products may also be contaminated with gluten (e.g., sauces, canned soups, broth, beer) [5]. The issue of gluten cross-contamination in naturally gluten-free (GF) food products is a serious concern for individuals with CD [6]. It occurs when a GF product comes into contact with a gluten-containing ingredient, which results in a product becoming unsafe for CD individuals, which may happen at any step of the food production process, from cultivation to final preparation, both at home and in a restaurant [7]. Another common source of cross-contamination is using the same kitchen utensils or surfaces for preparing dishes for individuals following a GF diet and those consuming gluten-containing products [8]. Moreover, individuals with CD may be unknowingly exposed to gluten, as it is also used as an additive in certain medications and cosmetics [4].

In the case of individuals with CD, the necessary recommendation is to follow a strict lifelong GF diet to prevent the symptoms and consequences of CD [9]. It means that CD patients must exclude all the products that may contain gluten or may be cross-contaminated with gluten from their diets in order to obtain a daily gluten intake below 10 mg a day, as indicated by the British Society of Gastroenterology as safe for CD patients [10], which was defined based on the previous studies [11]. So, in order to follow a GF diet, using GF products is necessary, and based on the Codex Alimentarius Standard for GF Foods, within this category of products are those characterized by a gluten content below 20 mg per 1 kg of product (20 ppm of gluten) [12].

It must be borne in mind that cereal-based GF products may be characterized by a lower nutritional value compared to their gluten-containing counterparts. For example, in the study of Bathrellou and Kontogianni [13], GF cereal products were found to contain a higher content of fat and saturated fatty acids, along with lower amounts of protein and iron. Therefore, it may be concluded that an inadequately balanced GF diet may negatively impact glucose and lipid metabolism and increase the risk of obesity and metabolic syndrome [14].

However, a relatively low adherence of CD patients to a GF diet is observed, as, in spite of their growing awareness, the adherence rate among adult CD patients is estimated to range from 42% to 91% [15]. This problem with a low adherence of CD patients to a GF diet results from a number of factors, including the high cost of GF products, their limited availability for CD patients, and their disputable quality [16].

Among the reasons for poor adherence, the high cost of GF products is commonly indicated as the most important. Depending on the study and country, it has been reported that GF products are 159% more expensive [17], 183% more expensive [18], 242% more expensive [19], or even 500% more expensive compared to standard non-GF products [20], while the relative price of GF products to non-GF products is still rising [21]. Such a situation is challenging, especially for lower-income families [22].

The low availability of GF products is often mentioned as a reason for non-adherence to the GF diet, which is indicated for 11% [23] or 15% of CD individuals [24]. Especially within a GF basic food basket (formed by the more economical products available), the availability is 42% lower compared to a regular basic food basket [20]. What is associated with a limited availability problem is the difference in the availability depending on the area, as in rural areas, the choice of products may be four times lower than in urban ones [25].

Last but not least, the problem is associated with the quality of GF products, as some products perceived by CD patients as GF and, therefore, safe may be gluten-contaminated, which was observed for more than 30% of assessed samples of GF-labeled products in the United States or India, as well as for more than 30% of naturally GF products in the United States, while the gluten content exceeded the limit of 20 ppm [4]. At the same time, the nutritional value of GF products is commonly lower compared to gluten-containing ones, which results from higher amounts of fats, trans fatty acids, and salt [26].

Taking into account the adherence problems described above, associated with the limited availability of GF products, their high cost, and commonly low quality, the aim of this study was to assess the frequency of buying and availability of GF cereal products in a population of Polish female CD patients.

## 2. Materials and Methods

### 2.1. Background Information

The presented cross-sectional study was a part of the international project administered by Harper Adams University in the United Kingdom, which was conducted in order to describe the availability of GF products for CD patients, as well as to describe their choices, beliefs, and opinions about those products [27,28]. The presented data were gathered and analyzed in Poland from May to August 2022 by the Institute of Human Nutrition Sciences, Warsaw University of Life Sciences (SGGW-WULS), Warsaw, Poland, after the questionnaire was disseminated by the Polish Celiac Society among the Society’s members.

This project was conducted based on the ethical approval of Harper Adams University, Newport, United Kingdom (No. 0439-202106-STAFF-CO_2_), and in agreement with the Declaration of Helsinki, while the study participants provided informed consent to participate.

### 2.2. Study Participants

This study was conducted in a population of Polish female CD patients, while the registered members of the Polish Celiac Society were invited to participate. The Polish Celiac Society is a member of the Association of European Celiac Societies (AOECS) [29], and it participates in conducting research on the CD population in Poland [30].

The applied inclusion criteria were as follows: membership in the Polish Celiac Society and providing informed consent for study participation. The exclusion criteria were as follows: CD not diagnosed (family members of CD patients being members of the Polish Celiac Society); male respondents; age under 18 years; living outside of Poland, as declared in the questionnaire; not purchasing GF products, as declared in the questionnaire.

The number of *n* = 978 respondents completed the questionnaire, but on the basis of the exclusion criteria, the following *n* = 431 were excluded: CD not diagnosed—*n* = 281; male respondents—*n* = 92; age of <18 years—*n* = 36; not living in Poland, declared within the questionnaire—*n* = 1; not purchasing GF products, declared within the questionnaire—*n* = 21. The final sample size was *n* = 547 respondents.

### 2.3. Applied Questionnaire and the Study Procedure

Within the conducted study, the same questionnaire was used as for the previous analysis conducted for a Polish population [27,28], as well as for the assessment conducted for the other countries [31], which allowed for obtaining comparable data for various countries, as presented within the previous study [32]. Within the previous studies, the applied questionnaire was presented in detail [27,28,31,32], while the Polish adaptation for the Polish GF food market and Polish population was described in a previous studies for the Polish population [27,28]. The standard questionnaire, as presented in the previous studies [27,28,31,32], was based on the previous study by Vriesekoop et al. [21]. The questionnaire was distributed, and the data were gathered using the computer-assisted web interview (CAWI) method while the target population, which consisted only of members of the Polish Celiac Society, was invited to participate, so the snowball effect did not occur.

The questionnaire applied in this study and described in the previous publications [27,28,31,32] covered the broader area, while in the presented study, the aim was associated only with GF cereal products. The following GF cereal product groups were included and listed within the questionnaire: bread; flour (for baking or cooking); pasta; Asian-style noodles; dumplings; wraps/tortillas; rice waffles; crackers; chips, crisps, puffs; puff pastry; cakes; biscuits/cookies; fried baked goods (e.g., donuts); breakfast cereals (e.g., cornflakes); oatmeal. The listed GF cereal product groups are in agreement with the products available in Poland [33].

The applied questionnaire was originally developed in English, and it was translated and adopted for the countries participating in this study, while the procedure of translation and adaptation for the novel language version was conducted in cooperation with the authors of the original questionnaire. Based on the recommendations by the World Health Organization (WHO) [34], the process of translation and adaptation to a Polish version included forward translation from English to Polish (conducted by a native Polish researcher being familiar with the discipline), backward translation from Polish to English (conducted by an independent translator with no knowledge of the questionnaire, being a native Polish researcher fluent in English), and the final polishing of the translated version, while keeping the conceptual, semantic, idiomatic, and cultural equivalence of the obtained tool (conducted by an expert panel of native Polish researchers being fluent in English), which is in agreement with the commonly applied procedure [35].

Participants of this study, the registered members of the Polish Celiac Society, received a link to the electronic version of the questionnaire, which was distributed by the Polish Celiac Society to the members. They completed a questionnaire regarding the frequency of buying specific GF cereal products, as well as about the problems with the availability of specific GF cereal products (2 independent questions, each with the same list of GF cereal products). For each product, the respondent was allowed to choose one of four potential categories of frequency: never, rarely, sometimes, often, and to choose one of two potential categories of problems: any problems or no problems.

The additional questions about problems with the availability of GF products on the Polish market and the quality of GF products on the Polish market were asked, while no problem with availability was declared by *n* = 16 respondents, so they were excluded from the analysis of the question about the problems with the availability of specific GF cereal products.

The other general questions that were asked allowed us to divide the study group into sub-groups to compare the frequency of buying and problems with the availability of GF cereal products in sub-groups of Polish female CD patients as follows:

(1) Age (single-choice closed-ended question to choose one of nine potential categories of age: <18 years, 18–24 years, 25–34 years, 35–44 years, 45–54 years, 55–64 years, 65–74 years, 75–84 years, and >84 years); after excluding respondents aged < 18, the others were aggregated into 2 sub-groups: age < 35 years (young adults), age ≥ 35 years (middle-aged and older adults), as no upper age limit was applied, which was in agreement of the categories of age presented by the other authors [36];
(2) Place of residence (open-ended question about the zip code)—after interpreting based on the zip code, participants were divided into 2 sub-groups: living in a big city (over 100,000 inhabitants—according to the Central Statistical Office in Poland equivalent to VI and VII class of the size of Polish cities [37]), living in a small town/village (less than 100,000 inhabitants), which was in agreement of the categories of place of residence presented by the other authors [38];
(3) Primary place of purchasing major grocery shopping (open-ended question about the names of the stores/markets)—after interpreting based on the names of the stores/markets, participants were divided into 2 sub-groups: purchasing in hypermarkets (over 2500 m^2^—according to the Central Statistical Office in Poland [39] and the trade press retail market analysis [40]), purchasing in shops other than hypermarkets (less than 2500 m^2^);
(4) Purchasing GF products online (single-choice closed-ended question to choose one of two potential categories of purchasing: purchasing GF products online at least occasionally, never purchasing GF products online)—participants were divided into 2 sub-groups: purchasing GF products online, never purchasing GF products online.

### 2.4. Statistical Analysis

The results were gathered as categorical data, presenting the frequencies and percentages for the frequency of buying and problems with the availability of GF cereal products in a population of Polish female CD patients stratified into sub-groups. The comparison of the sub-groups was conducted while using the chi^2^ test.

The value of *p* ≤ 0.05 was indicated as a statistically significant difference between sub-groups. The statistical analysis was conducted while using Statistica 8.0 (Statsoft Inc., Tulsa, OK, USA).

## 3. Results

Table 1 presents the characteristics of the studied group of female CD patients. The age distribution shows that the majority of participants were between 25 and 44 years old (72.6%), with the largest group aged 25–34 years (37.1%). For the place of residence, almost an equal share of participants represented big cities (51.0%) and small towns or villages (49.0%). Most women reported carrying out their major shopping in hypermarkets (72.6%) and purchasing GF products online at least occasionally (81.4%). In the studied group of CD patients, the availability of GF products was indicated as a problem by nearly all respondents (97.1%), but the quality of GF products was also indicated as a problem by a vast majority of the group (63.6%).

Table 2 presents the declared frequency of buying specific GF cereal products in the studied group of female CD patients. The majority of the studied female CD patients declared often purchasing GF cereal products such as flour for baking or cooking (62.6%), pasta (61.0%), and bread (59.6%). A little bit less frequently, the studied female CD patients were buying such GF cereal products as rice waffles (68.8% of respondents declaring that they buy it sometimes or often), chips, crisps, and puffs (61.6%), breakfast cereals (57.5%), oatmeal (56.2%), and biscuits/cookies (55.7%). Less often, the studied female CD patients declared buying GF cereal products as Asian-style noodles (67.1% of respondents declared that they bought it rarely or never), wraps/tortillas (80%), and cakes (83.2%). At the same time, the majority of the studied female CD patients declared never purchasing GF cereal products such as puff pastry (65.9%), fried or baked goods (63.3%), dumplings (54.7%), and crackers (51.1%).

Table 3 presents declared problems with the availability of specific GF cereal products in the sub-group of female CD patients declaring a general problem with the availability of GF products (*n* = 531). The only product for which the majority of the studied sub-group of female CD patients declared problems with availability was dumplings (51.8%), while for the other products, over 50% declared no problems with availability. The highest share of participants declaring no problems with availability was stated for rice waffles (97.7%), breakfast cereals (94.0%), chips, crisps, puffs (92.8%), Asian-style noodles (89.3%), crackers (75.9%), biscuits/cookies (75.7%), and oatmeal (75.5%).

Table 4 presents the declared frequency of buying specific GF cereal products in the studied group of female CD patients, stratified by age. For a number of products, younger respondents declared a higher frequency of buying them often compared to older respondents, which was observed for products such as pasta (68% vs. 55%, *p* = 0.0073), chips, crisps, puffs (33% vs. 15%, *p* < 0.0001), and Asian-style noodles (19% vs. 11%, *p* = 0.0269). At the same time, older respondents declared a higher frequency of buying flour compared to younger respondents (67% vs. 58%, *p* = 0.0358); as well, they declared a higher frequency of never buying wraps/tortillas compared to younger respondents (49% vs. 38%, *p* = 0.0181).

Table 5 presents the declared frequency of buying specific GF cereal products in the studied group of female CD patients, stratified by place of residence. Overall, the purchasing behavior of participants living in big cities and small towns/villages showed no statistically significant differences.

Table 6 presents the declared frequency of buying specific GF cereal products in the studied group of female CD patients, stratified by the primary place of grocery shopping. For biscuits/cookies, respondents purchasing major grocery shopping in hypermarkets declared a higher frequency of buying them often compared to those purchasing in other shops (21% vs. 9%, *p* = 0.0039). At the same time, respondents purchasing in other shops declared a higher frequency of never buying some products compared to respondents purchasing in hypermarkets, which was observed for puff pastry (72% vs. 63%, *p* = 0.0076), dumplings (64% vs. 49%, *p* = 0.0002), and wraps/tortillas (54% vs. 40%, *p* = 0.0038).

Table 7 presents the declared frequency of buying specific GF cereal products in the studied group of female CD patients, stratified by purchasing GF products online. Overall, the purchasing behavior of participants purchasing GF products online or not showed no statistically significant differences for most of the products. Only for fried or baked goods, respondents who have never purchased GF products online declared a higher frequency of never buying them compared to respondents purchasing online at least occasionally (74% vs. 61%, *p* = 0.0284).

Table 8 presents the declared problems with the availability of specific GF cereal products in the sub-group of female CD patients, declaring a general problem with the availability of GF products, stratified by age. For a number of products, a higher share of younger respondents declared problems with availability compared to older respondents, which was observed for the products such as wraps/tortillas (52% vs. 34%, *p* = 0.0001), puff pastry (49% vs. 40%, *p* = 0.0294), fried baked goods (49% vs. 34%, *p* = 0.0008), biscuits/cookies (29% vs. 20%, *p* = 0.0148), and Asian-style noodles (15% vs. 7%, *p* = 0.0046).

Table 9 presents the declared problems with availability of specific GF cereal products in the sub-group of female CD patients declaring general problems with availability of GF products, stratified by place of residence. Overall, the problems with availability declared by participants living in big cities and small towns/villages showed no statistically significant differences for most of the products. Only for pasta, a higher share of respondents living in big cities declared problems with availability compared to respondents living in small towns/villages (47% vs. 37%, *p* = 0.0245).

Table 10 presents the declared problems with the availability of specific GF cereal products in the sub-group of female CD patients declaring general problems with the availability of GF products, stratified by primary place of purchasing major grocery shopping. A higher share of respondents purchasing in other shops than hypermarkets declared problems with the availability of some products compared to respondents purchasing in hypermarkets, which was observed for puff pastry (53% vs. 41%, *p* = 0.0246), biscuits/cookies (36% vs. 20%, *p* = 0.0002), and breakfast cereals (12% vs. 4%, *p* = 0.0011).

Table 11 presents the declared problems with availability of specific GF cereal products in the sub-group of female CD patients declaring general problems with the availability of GF products, stratified by purchasing GF products online. Overall, the problems with availability declared by participants purchasing GF products online and those who refrained from purchasing them online showed no statistically significant differences for most of the products. Only for wraps/tortillas, a higher share of respondents purchasing online at least occasionally declared problems with availability compared to respondents who have never purchased GF products online (45% vs. 33%, *p* = 0.0411).

**Figure 1:**
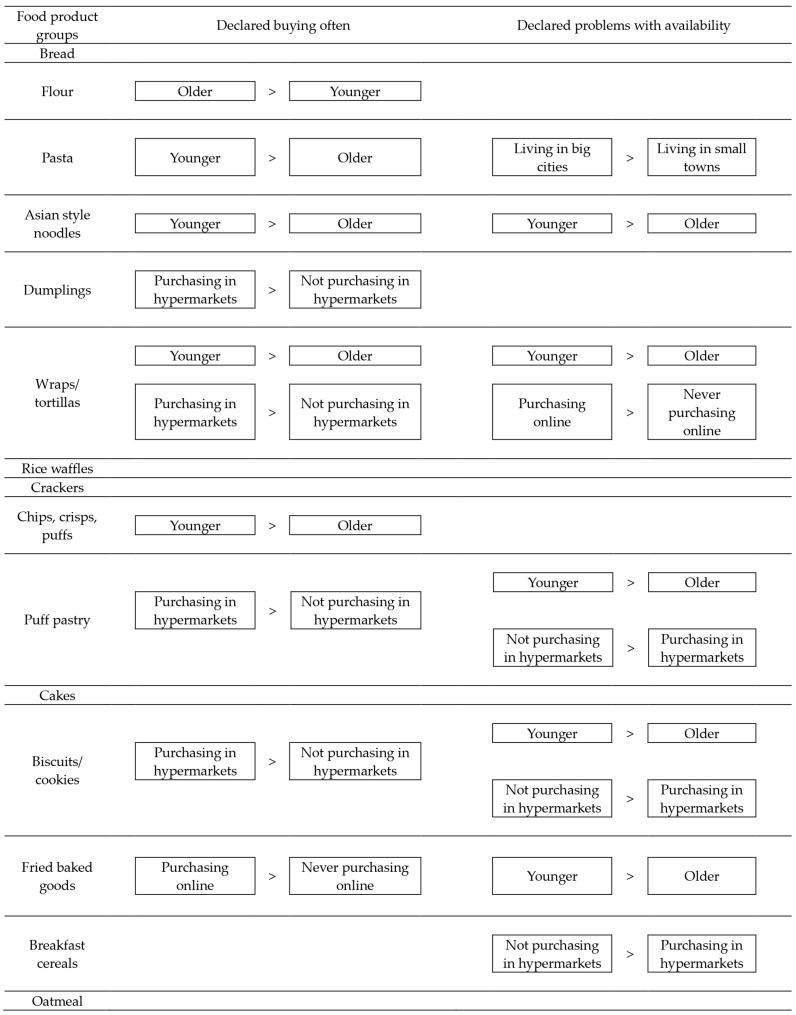
The graphical summary of the results of the frequency of buying and problems with the availability of the specific gluten-free (GF) cereal products in the studied group of female celiac disease (CD) patients.

The graphical summary of the results of the frequency of buying and problems with the availability of the specific GF cereal products in the studied group of female CD patients is presented in Figure 1. It was observed that the agreement of the observations was stated for Asian-style noodles and wraps/tortillas, whereas for younger participants, both the higher frequency of buying and the higher problems with availability were stated compared to the older participants. Moreover, for puff pastry and biscuits/cookies, individuals purchasing in hypermarkets declared a higher frequency of buying compared to those not purchasing in hypermarkets, while individuals not purchasing in hypermarkets declared higher problems with availability.

Table 12 presents the declared problems with the availability of specific GF cereal products in the sub-group of female CD patients purchasing specific products, stratified by the frequency of buying them. For a number of products, a lower share of respondents purchasing them rarely declared the problems with availability compared to the respondents purchasing them more often, which was observed for products such as wraps/tortillas (45% vs. 48% vs. 82%, *p* = 0.0126), bread (29% vs. 39% vs. 38%, *p* < 0.0001), and Asian-style noodles (7% vs. 12% vs. 21%, *p* = 0.0148). At the same time, for cakes, a lower share of respondents purchasing them often declared problems with availability compared to respondents purchasing them less often (21% vs. 52% vs. 30%, *p* = 0.0018).

## 4. Discussion

The presented study provides insight into the purchasing behaviors, including frequency of buying and perceived availability of GF cereal products in a population of Polish female CD patients. As observed, age, primary place of purchasing major grocery shopping, and purchasing GF products online were the important determinants of the frequency of buying, but there was no influence on the place of residence. At the same time, for the perceived availability of GF cereal products, all the studied factors were determinants.

For age, it may be stated that younger Polish female CD patients, more often than older ones, were purchasing GF products such as pasta, chips, crisps and puffs, Asian-style noodles, and wraps/tortillas, while older individuals, more often than younger ones, purchased GF flour. It can be assumed that the older individuals used the GF flour to prepare the other GF products instead of trying to purchase them. It was indicated by Šmídová and Rysová [41] that due to the limited shelf life of GF products, the home baking of GF bread and preparing other GF products at home may be chosen [42]. Moreover, the other problems of the GF products market, including unsatisfactory availability, high prices, and low nutritional value [21], may be the other issues causing participants to choose to prepare their own GF products instead of purchasing them. The results of the conducted study indicated that such a situation may be typical, especially for older CD patients. In the presented study, older CD patients declared either similar or lower levels of problems with the availability of GF products (but not a higher one), which may correspond with preparing them on their own, as the availability of GF products for CD patients is particularly important, as these foods are consumed daily, and their unavailability could disrupt daily life routines [22]. Moreover, the fact that older CD patients less often than younger ones reported problems with the availability of GF snacks, such as puff pastry, fried or baked goods, or biscuits/cookies, as well as some GF products typical for other ethnic cuisines, such as wraps/tortillas, or Asian-style noodles, may result from the lower interest in such products. This situation may reflect variations in dietary preferences, nutritional knowledge, as well as economic limitations. It corresponds with a lower intake of snacks in older individuals compared to younger ones [43] and a lower willingness to pay for ethnic products in older individuals compared to younger ones [44]. Younger individuals may have greater exposure to modern dietary trends and may be more familiar with online shopping [45], whereas older individuals tend to be more self-sufficient in food preparation, relying on their cooking skills and often better kitchen facilities [46].

For the primary place of purchasing major grocery shopping, it may be stated that Polish female CD patients buying in hypermarkets more often than the other patients purchased GF products such as biscuits/cookies, puff pastry, dumplings, and wraps/tortillas. Taking this into account, the hypermarkets may be indicated as a potential place with better availability and choice of such products, which corresponds with the results of the studies by other authors, indicating a good availability of GF products in regular and quality supermarkets as well as online [47], with almost no GF products being available in corner shops [48]. If the higher availability of GF products in hypermarkets is supposed, the higher frequency of declared problems with the availability of GF puff pastry, biscuits/cookies, and breakfast cereals, in the case of the CD patients not buying GF products in hypermarkets, is not surprising.

However, one important observation must be made regarding the declared problems with the availability of GF products. It seems that the declared problems are probably associated with two diverse mechanisms—either frequent purchasing (as individuals not purchasing may not be interested in such a product at all) or rare purchasing (which may result from poor availability). The first mechanism was observed for young Polish female CD patients— often purchasing Asian-style noodles and wraps/tortillas, but at the same time declaring problems with their availability. The second mechanism was observed for female CD patients buying GF products in places other than hypermarkets—rarely purchasing biscuits/cookies and puff pastry, and at the same time declaring problems with their availability. It indicates that there is still potential to broaden the GF products market, in spite of the fact that GF products are of a higher price than regular ones [49], the GF market is increasing [50], and it should be increasing to meet the expectations of CD patients.

For purchasing GF products online, it may be stated that the observed differences between subgroups were rather random, as within a sub-group of respondents not purchasing GF products online, a higher share declared never buying GF fried baked goods compared to respondents purchasing online, as well as a lower share declaring problems with availability of GF wraps/tortillas. So, it may be supposed that respondents purchasing GF products online may be interested in a higher diversity of GF products and may obtain them while searching for GF products online. However, the other problem with this issue may be the ability and willingness to shop online, as some respondents prefer offline shopping, where they can inspect products directly and receive personalized assistance from sales staff [51]. It is commonly indicated that, especially for older individuals who may experience marginalization and social exclusion [52], assistance from sales staff can greatly enhance the shopping experience and information exchange [51].

For the place of residence, interestingly, there was no influence on the frequency of buying the specific GF products, which may result from the fact that even respondents living in rural areas, while searching for GF products, may obtain them from remote hypermarkets or online. It corresponds with the results of the previous study [27], as CD patients living in small towns/villages were even more satisfied with GF products’ quality than those living in big cities, which was associated with the fact that they had to actively search for them, and sometimes, they had to travel to another city to buy the GF products they needed. Similarly, in the presented study, CD patients living in big cities declared more problems with the availability of GF pasta compared to respondents living in small towns/villages. This allows us to state that, in general, living in a big city or a small town/village does not influence the declared availability of most GF products. The systematic review by Hall et al. [15] on adherence to a GF diet in adults emphasized that while the country or region of residence appears to be important in terms of the availability of GF products, the rural or urban area has not been explicitly examined so far.

At the same time, it must be indicated that the limited choice or low availability of GF products may lead to a lack of variety in purchased GF products, low dietary diversity of chosen products, as well as a low diversity of a GF diet, which consequently may negatively impact diet quality. Dietary diversity is crucial for maintaining overall diet quality, as it is associated with improved nutrient intake, better health outcomes, and a wider variety of foods and food groups within the recommended standards, leading to an increased intake of essential nutrients [53]. As shown, corn, rice, and potatoes are starch products most commonly consumed by CD patients [54]. Incorporating other GF cereals, such as buckwheat, millet, quinoa, or oat, would improve the diet’s diversity. However, based on the results of the Canadian study, it can be assumed that the conventional oat is heavily contaminated with gluten from other grains [55]. Similarly, in the American study, it was found that 75% of oat samples labeled as GF were contaminated with gluten [56]. According to the recommendations by the Polish Celiac Society, CD patients must not consume conventional oats due to gluten contamination but only confirmed GF ones [57]. On the other hand, GF oat products are not well tolerated by all CD patients (approximately 5% of the participants do not tolerate them at all) and must be included in the diet with extra precaution [57].

These challenges underline the need for a more reliable and accessible supply of GF products for all CD patients. The increasing demand for GF products is transforming the GF market into a significant sector of the global food industry. Between 2018 and 2022, the global demand for GF products grew by approximately 16% [58]. It should be noted that this is due to the fact that even individuals without diagnosed CD or other gluten issues are trying to follow a GF diet to improve certain aspects of their lives, even though there is evidence indicating that there are significant drawbacks to following a GF diet without a medical reason [5]. In spite of the fact that a recent study by Dean et al. [32], which collected data from 13 countries, showed that GF product assortments have improved; it also highlights that availability is still a barrier, especially for individuals who must strictly adhere to a GF diet, such as CD patients. As highlighted in the systematic review by Hall et al. [15], participants with CD who are adequately supported tend to have higher adherence rates to a GF diet. So, understanding the various factors influencing their purchasing behavior and providing an adequate choice of GF products may help to enhance their satisfaction and health outcomes. This is particularly important for public health, as increased access to suitable GF products can support better disease management and quality of life for CD patients.

In spite of the important observations, it is worth noting that there are some limitations of this study associated with its cross-sectional design, which only provided data from one point in time and does not allow for observing long-term trends. The tool could not have been fully validated due to the nature of the questions; the questionnaire assessed only declared behaviors rather than actual practices, which may not accurately reflect participants’ true purchasing habits. As a result, the findings are based on self-reported data, which can be influenced by recall bias or social desirability bias, leading to potential discrepancies between declared and actual behaviors.

## 5. Conclusions

The population of Polish female CD patients shows significant variability in terms of the chosen GF cereal products with age, primary place of purchasing major grocery shopping and purchasing GF products online, but not the place of residence, as the major determinants.

The reported problems regarding the availability of GF products in Polish female CD patients can be linked to two diverse mechanisms—either frequent purchasing (as individuals not purchasing may not be interested in such products at all) or rare purchasing (which may result from poor availability). Addressing these issues by increasing the availability of a wider range of GF cereal products could help improve the dietary diversity of Polish female CD patients, ultimately supporting better disease management and general health.

## Figures and Tables

**Table 1:** Characteristics of the studied group of female celiac disease (CD) patients (*n* = 547).

Variable	Categories	*n* (%)
Age (years)	18–24	52 (9.5%)
	25–34	203 (37.1%)
	35–44	194 (35.5%)
	45–54	75 (13.7%)
	55–64	18 (3.3%)
	65–74	4 (0.7%)
	75-84	1 (0.2%)
Place of residence	Big city (>100,000 inhabitants)	279 (51.0%)
	Small town/village (<100,000 inhabitants)	268 (49.0%)
Major grocery shopping	Hypermarkets	397 (72.6%)
	Other	150 (27.4%)
GF products purchased online	At least occasionally	445 (81.4%)
	Never	102 (18.6%)
Problem with availability of GF products	Problems confirmed	531 (97.1%)
	No problems	16 (2.9%)
Problem with quality of GF products	Problems confirmed	348 (63.6%)
	No problems	199 (36.4%)

GF—gluten-free.

**Table 2:** Declared frequency of buying specific gluten-free (GF) cereal products in the studied group of female celiac disease (CD) patients (*n* = 547).

Gluten-Free (GF) Cereal Products	Never	Rarely	Sometimes	Often
Bread (*n* = 547)	22 (4.0%)	104 (19.0%)	95 (17.4%)	326 (59.6%)
Flour (for baking or cooking) (*n* = 546)	18 (3.3%)	57 (10.4%)	129 (23.6%)	342 (62.6%)
Pasta (*n* = 543)	9 (1.7%)	44 (8.1%)	159 (29.3%)	331 (61.0%)
Asian-style noodles (*n* = 525)	234 (44.6%)	118 (22.5%)	95 (18.1%)	78 (14.9%)
Dumplings (*n* = 541)	296 (54.7%)	173 (32.0%)	58 (10.7%)	14 (2.6%)
Wraps/tortillas (*n* = 540)	237 (43.9%)	195 (36.1%)	90 (16.7%)	18 (3.3%)
Rice waffles (*n* = 542)	49 (9.0%)	120 (22.1%)	171 (31.5%)	202 (37.3%)
Crackers (*n* = 540)	276 (51.1%)	181 (33.5%)	75 (13.9%)	8 (1.5%)
Chips, crisps, puffs (*n* = 542)	67 (12.4%)	141 (26.0%)	208 (38.4%)	126 (23.2%)
Puff pastry (*n* = 545)	359 (65.9%)	152 (27.9%)	30 (5.5%)	4 (0.7%)
Cakes (*n* = 534)	215 (40.3%)	229 (42.9%)	76 (14.2%)	14 (2.6%)
Biscuits/cookies (*n* = 546)	54 (9.9%)	188 (34.4%)	208 (38.1%)	96 (17.6%)
Fried baked goods (e.g., donuts) (*n* = 545)	345 (63.3%)	153 (28.1%)	44 (8.1%)	3 (0.6%)
Breakfast cereals (e.g., cornflakes) (*n* = 546)	94 (17.2%)	138 (25.3%)	171 (31.3%)	143 (26.2%)
Oatmeal (*n* = 542)	131 (24.2%)	106 (19.6%)	127 (23.4%)	178 (32.8%)

**Table 3:** Declared problems with availability of specific gluten-free (GF) cereal products in the sub-group of female celiac disease (CD) patients declaring general problems with availability of GF products (*n* = 531).

Gluten-Free (GF) Cereal Products (*n* = 531)	Declared Problems	No Problems Declared
Bread	189 (35.6%)	342 (64.4%)
Flour (for baking or cooking)	195 (36.7%)	336 (63.3%)
Pasta	222 (41.8%)	309 (58.2%)
Asian-style noodles	57 (10.7%)	474 (89.3%)
Dumplings	275 (51.8%)	256 (48.2%)
Wraps/tortillas	225 (42.4%)	306 (57.6%)
Rice waffles	12 (2.3%)	519 (97.7%)
Crackers	128 (24.1%)	403 (75.9%)
Chips, crisps, puffs	38 (7.2%)	493 (92.8%)
Puff pastry	236 (44.4%)	295 (55.6%)
Cakes	167 (31.5%)	364 (68.5%)
Biscuits/cookies	129 (24.3%)	402 (75.7%)
Fried baked goods (e.g., donuts)	218 (41.1%)	313 (58.9%)
Breakfast cereals (e.g., cornflakes)	32 (6.0%)	499 (94.0%)
Oatmeal	130 (24.5%)	401 (75.5%)

**Table 4:** Declared frequency of buying specific gluten-free (GF) cereal products in the studied group of female celiac disease (CD) patients (*n* = 547), stratified by age.

Gluten-Free (GF) Cereal Products	Age < 35			Age ≥ 35			*p*
	Never	Rarely/Sometimes	Often	Never	Rarely/Sometimes	Often	
Bread (*n* = 255/292)	7 (2.8%)	83 (32.5%)	165 (64.7%)	15 (5.1%)	116 (39.7%)	161 (55.2%)	0.0509
Flour (for baking or cooking) (*n* = 255/291)	7 (2.8%)	101 (39.6%)	147 (57.6%)	11 (3.8%)	85 (29.2%)	195 (67.0%)	0.0358
Pasta (*n* = 253/290)	5 (2.0%)	77 (30.4%)	171 (67.6%)	4 (1.4%)	126 (43.4%)	160 (55.2%)	0.0073
Asian-style noodles (*n* = 249/276)	100 (40.1%)	102 (41.0%)	47 (18.9%)	134 (48.6%)	111 (40.2%)	31 (11.2%)	0.0269
Dumplings (*n* = 254/287)	136 (53.5%)	110 (43.3%)	8 (3.2%)	160 (55.7%)	121 (42.2%)	6 (2.1%)	0.6889
Wraps/tortillas (*n* = 252/288)	95 (37.7%)	146 (57.9%)	11 (4.4%)	142 (49.3%)	139 (48.3%)	7 (2.4%)	0.0181
Rice waffles (*n* = 253/289)	24 (9.5%)	128 (50.6%)	101 (39.9%)	25 (8.7%)	163 (56.4%)	101 (34.9%)	0.3971
Crackers (*n* = 251/289)	130 (51.8%)	115 (45.8%)	6 (2.4%)	146 (50.5%)	141 (48.8%)	2 (0.7%)	0.2336
Chips, crisps, puffs (*n* = 253/289)	21 (8.3%)	149 (58.9%)	83 (32.8%)	46 (15.9%)	200 (69.2%)	43 (14.9%)	<0.0001
Puff pastry (*n* = 255/290)	164 (64.3%)	88 (34.5%)	3 (1.2%)	195 (67.2%)	94 (32.5%)	1 (0.3%)	0.4418
Cakes (*n* = 250/284)	95 (38.0%)	149 (59.6%)	6 (2.4%)	120 (42.3%)	156 (54.9%)	8 (2.8%)	0.5506
Biscuits/cookies (*n* = 254/292)	23 (9.1%)	184 (72.4%)	47 (18.5%)	31 (10.6%)	212 (72.6%)	49 (16.8%)	0.7540
Fried baked goods (e.g., donuts) (*n* = 253/292)	163 (64.4%)	88 (34.8%)	2 (0.8%)	182 (62.3%)	109 (37.3%)	1 (0.4%)	0.6598
Breakfast cereals (e.g., cornflakes) (*n* = 255/291)	38 (14.9%)	147 (57.6%)	70 (27.5%)	56 (19.2%)	162 (55.7%)	73 (25.1%)	0.3921
Oatmeal (*n* = 254/288)	72 (28.3%)	101 (39.8%)	81 (31.9%)	59 (20.5%)	132 (45.8%)	97 (33.7%)	0.0936

**Table 5:** Declared frequency of buying specific gluten-free (GF) cereal products in the studied group of female celiac disease (CD) patients (*n* = 547), stratified by place of residence.

Gluten-Free (GF) Cereal Products	Living in a Big City			Living in a Small Town/Village			*p*
	Never	Rarely/Sometimes	Often	Never	Rarely/Sometimes	Often	
Bread (*n* = 279/268)	12 (4.3%)	100 (35.8%)	167 (59.9%)	10 (3.7%)	99 (36.9%)	159 (59.4%)	0.9222
Flour (for baking or cooking) (*n* = 278/268)	10 (3.6%)	103 (37.0%)	165 (59.4%)	8 (3.0%)	83 (31.0%)	177 (66.0%)	0.2710
Pasta (*n* = 278/265)	4 (1.5%)	106 (38.1%)	168 (60.4%)	5 (1.9%)	97 (36.6%)	163 (61.5%)	0.8717
Asian-style noodles (*n* = 268/257)	117 (43.7%)	115 (42.9%)	36 (13.4%)	117 (45.6%)	98 (38.1%)	42 (16.3%)	0.4519
Dumplings (*n* = 275/266)	138 (50.2%)	128 (46.5%)	9 (3.3%)	158 (59.4%)	103 (38.7%)	5 (1.9%)	0.0800
Wraps/tortillas (*n* = 276/264)	111 (40.2%)	155 (56.2%)	10 (3.6%)	126 (47.7%)	130 (49.3%)	8 (3.0%)	0.2123
Rice waffles (*n* = 277/265)	26 (9.4%)	141 (50.9%)	110 (39.7%)	23 (8.7%)	150 (56.6%)	92 (34.7%)	0.4063
Crackers (*n* = 278/262)	141 (50.7%)	132 (47.5%)	5 (1.8%)	135 (51.5%)	124 (47.3%)	3 (1.2%)	0.8160
Chips, crisps, puffs (*n* = 277/265)	34 (12.3%)	183 (66.1%)	60 (21.6%)	33 (12.5%)	166 (62.6%)	66 (24.9%)	0.6494
Puff pastry (*n* = 278/267)	176 (63.3%)	101 (36.3%)	1 (0.4%)	183 (68.5%)	81 (30.3%)	3 (1.2%)	0.2108
Cakes (*n* = 273/261)	100 (36.6%)	165 (60.5%)	8 (2.9%)	115 (44.1%)	140 (53.6%)	6 (2.3%)	0.2108
Biscuits/cookies (*n* = 279/267)	24 (8.6%)	209 (74.9%)	46 (16.5%)	30 (11.2%)	187 (70.0%)	50 (18.8%)	0.4081
Fried baked goods (e.g., donuts) (*n* = 278/267)	174 (62.6%)	102 (36.7%)	2 (0.7%)	171 (64.0%)	95 (35.6%)	1 (0.4%)	0.8244
Breakfast cereals (e.g., cornflakes) (*n* = 278/268)	48 (17.3%)	163 (58.6%)	67 (24.1%)	46 (17.2%)	146 (54.4%)	76 (28.4%)	0.5062
Oatmeal (*n* = 277/265)	63 (22.7%)	114 (41.2%)	100 (36.1%)	68 (25.7%)	119 (44.9%)	78 (29.4%)	0.2525

**Table 6:** Declared frequency of buying specific gluten-free (GF) cereal products in the studied group of female celiac disease (CD) patients (*n* = 547), stratified by primary place of purchasing major grocery shopping.

Gluten-Free (GF) Cereal Products	Purchasing in Hypermarkets			Purchasing in Other Shops than Hypermarkets			*p*
	Never	Rarely/Sometimes	Often	Never	Rarely/Sometimes	Often	
Bread (*n* = 397/150)	18 (4.5%)	244 (34.0%)	244 (61.5%)	4 (2.7%)	64 (42.6%)	82 (54.7%)	0.1332
Flour (for baking or cooking) (*n* = 396/150)	10 (2.5%)	243 (36.1%)	243 (61.4%)	8 (5.3%)	43 (28.7%)	99 (66.0%)	0.0932
Pasta (*n* = 395/148)	7 (1.8%)	239 (37.7%)	239 (60.5%)	2 (1.4%)	54 (36.4%)	92 (62.2%)	0.9015
Asian-style noodles (n = 379/146)	175 (46.2%)	52 (40.1%)	52 (13.7%)	59 (40.4%)	61 (41.8%)	26 (17.8%)	0.3599
Dumplings (*n* = 394/147)	194 (49.2%)	11 (48.0%)	11 (2.8%)	102 (69.4%)	42 (28.6%)	3 (2.0%)	0.0002
Wraps/tortillas (*n* = 392/148)	157 (40.1%)	11 (57.1%)	11 (2.8%)	80 (54.1%)	61 (41.2%)	7 (4.7%)	0.0038
Rice waffles (*n* = 393/149)	34 (8.7%)	149 (53.4%)	149 (37.9%)	15 (10.1%)	81 (54.4%)	53 (35.5%)	0.8122
Crackers (*n* = 391/149)	200 (51.2%)	5 (47.5%)	5 (1.3%)	76 (51.0%)	70 (47.0%)	3 (2.0%)	0.8182
Chips, crisps, puffs (*n* = 393/149)	52 (13.2%)	93 (63.1%)	93 (23.7%)	15 (10.1%)	101 (67.8%)	33 (22.1%)	0.5092
Puff pastry (*n* = 396/149)	251 (63.3%)	1 (36.4%)	1 (0.3%)	108 (72.5%)	38 (25.5%)	3 (2.0%)	0.0076
Cakes (*n* = 388/146)	155 (39.9%)	13 (56.7%)	13 (3.4%)	60 (41.1%)	85 (58.2%)	1 (0.7%)	0.2285
Biscuits/cookies (*n* = 397/149)	38 (9.6%)	83 (69.5%)	83 (20.9%)	16 (10.8%)	120 (80.5%)	13 (8.7%)	0.0039
Fried baked goods (e.g., donuts) (*n* = 396/149)	249 (62.8%)	3 (36.4%)	3 (0.8%)	96 (64.0%)	53 (35.3%)	1 (0.7%)	0.5519
Breakfast cereals (e.g., cornflakes) (*n* = 396/150)	69 (17.4%)	103 (56.6%)	103 (26.0%)	25 (16.7%)	85 (56.6%)	40 (26.7%)	0.9732
Oatmeal (*n* = 395/147)	94 (23.8%)	126 (44.3%)	126 (31.9%)	37 (25.2%)	58 (39.4%)	52 (35.4%)	0.5877

**Table 7:** Declared frequency of buying specific gluten-free (GF) cereal products in the studied group of female celiac disease (CD) patients (*n* = 547), stratified by purchasing GF products online.

Gluten-Free (GF) Cereal Products	GF Products Purchased Online			GF Products Never Purchased Online			*p*
	Never	Rarely/Sometimes	Often	Never	Rarely/Sometimes	Often	
Bread (*n* = 445/102)	20 (4.5%)	256 (38.0%)	256 (57.5%)	2 (2.0%)	30 (29.4%)	70 (68.6%)	0.0947
Flour (for baking or cooking) (*n* = 444/102)	17 (3.8%)	282 (32.7%)	282 (63.5%)	1 (1.0%)	41 (40.2%)	60 (58.8%)	0.1560
Pasta (*n* = 441/102)	7 (1.6%)	279 (35.1%)	279 (63.3%)	2 (2.0%)	48 (47.0%)	52 (51.0%)	0.0719
Asian-style noodles (*n* = 430/95)	187 (43.5%)	64 (41.6%)	64 (14.9%)	47 (49.5%)	34 (35.8%)	14 (14.7%)	0.5272
Dumplings (*n* = 439/102)	233 (53.1%)	14 (43.7%)	14 (3.2%)	63 (61.8%)	39 (38.2%)	0 (0.0%)	0.0828
Wraps/tortillas (*n* = 438/102)	185 (42.2%)	14 (54.6%)	14 (3.2%)	52 (51.0%)	46 (45.1%)	4 (3.9%)	0.2257
Rice waffles (*n* = 440/102)	36 (8.2%)	166 (54.1%)	166 (37.7%)	13 (12.7%)	53 (52.0%)	36 (35.3%)	0.3484
Crackers (*n* = 438/102)	220 (50.3%)	8 (47.9%)	8 (1.8%)	56 (54.9%)	46 (45.1%)	0 (0.0%)	0.3076
Chips, crisps, puffs (*n* = 440/102)	48 (10.9%)	103 (65.7%)	103 (23.4%)	19 (18.7%)	60 (58.8%)	23 (22.5%)	0.0992
Puff pastry (*n* = 443/102)	287 (64.8%)	4 (34.3%)	4 (0.9%)	72 (70.6%)	30 (29.4%)	0 (0.0%)	0.3789
Cakes (*n* = 435/99)	175 (40.2%)	13 (56.8%)	13 (3.0%)	40 (40.4%)	58 (58.6%)	1 (1.0%)	0.5351
Biscuits/cookies (*n* = 444/102)	39 (8.8%)	80 (73.2%)	80 (18.0%)	15 (14.7%)	71 (69.6%)	16 (15.7%)	0.1878
Fried baked goods (e.g., donuts) (*n* = 443/102)	269 (60.7%)	3 (38.6%)	3 (0.7%)	76 (74.5%)	26 (25.5%)	0 (0.0%)	0.0284
Breakfast cereals (e.g., cornflakes) (*n* = 444/102)	72 (16.2%)	119 (57.0%)	119 (26.8%)	22 (21.6%)	56 (54.9%)	24 (23.5%)	0.4101
Oatmeal (*n* = 442/100)	102 (23.1%)	153 (42.3%)	153 (34.6%)	29 (29.0%)	46 (46.0%)	25 (25.0%)	0.1543

**Table 8:** Declared problems with availability of specific gluten-free (GF) cereal products in the sub-group of female celiac disease (CD) patients declaring general problems with availability of GF products (*n* = 531), stratified by age.

Gluten-Free (GF) Cereal Products	Age < 35 (*n* = 251)		Age ≥ 35 (*n* = 280)		*p*
	Declared Problems	No Problems Declared	Declared Problems	No Problems Declared	
Bread	91 (36.3%)	160 (63.7%)	98 (35.0%)	182 (65.0%)	0.7630
Flour (for baking or cooking)	94 (37.5%)	157 (62.5%)	101 (36.1%)	179 (63.9%)	0.7421
Pasta	104 (41.4%)	147 (58.6%)	118 (42.1%)	162 (57.9%)	0.8687
Asian-style noodles	37 (14.7%)	214 (85.3%)	20 (7.1%)	260 (92.9%)	0.0046
Dumplings	140 (55.8%)	111 (44.2%)	135 (48.2%)	145 (51.8%)	0.0815
Wraps/tortillas	130 (51.8%)	121 (48.2%)	95 (33.9%)	185 (66.1%)	0.0001
Rice waffles	5 (2.0%)	246 (98.0%)	7 (2.5%)	273 (97.5%)	0.6933
Crackers	68 (27.1%)	183 (72.9%)	60 (21.4%)	220 (78.6%)	0.1279
Chips, crisps, puffs	22 (8.8%)	229 (91.2%)	16 (5.7%)	264 (94.3%)	0.1734
Puff pastry	124 (49.4%)	127 (50.6%)	112 (40.0%)	168 (60.0%)	0.0294
Cakes	89 (35.5%)	162 (64.5%)	78 (27.9%)	202 (72.1%)	0.0597
Biscuits/cookies	73 (29.1%)	178 (70.9%)	56 (20.0%)	224 (80.0%)	0.0148
Fried baked goods (e.g., donuts)	122 (48.6%)	129 (51.4%)	96 (34.3%)	184 (65.7%)	0.0008
Breakfast cereals (e.g., cornflakes)	13 (5.2%)	238 (94.8%)	19 (6.8%)	261 (93.2%)	0.4357
Oatmeal	60 (23.9%)	191 (76.1%)	70 (25.0%)	210 (75.0%)	0.7693

**Table 9:** Declared problems with availability of specific gluten-free (GF) cereal products in the sub-group of female celiac disease (CD) patients declaring general problems with availability of GF products (*n* = 531), stratified by place of residence.

Gluten-Free (GF) Cereal Products	Living in a Big City (*n* = 272)		Living in a Small Town/Village (*n* = 272/259)		*p*
	Declared Problems	No Problems Declared	Declared Problems	No Problems Declared	
Bread	103 (37.9%)	(62.1%)	86 (33.2%)	173 (66.8%)	0.3025
Flour (for baking or cooking)	102 (37.5%)	(62.5%)	93 (35.9%)	166 (64.1%)	0.7719
Pasta	127 (46.7%)	(53.3%)	95 (36.7%)	164 (63.3%)	0.0245
Asian-style noodles	30 (11.0%)	(89.0%)	27 (10.4%)	232 (89.6%)	0.9333
Dumplings	138 (50.7%)	(49.3%)	137 (52.9%)	122 (47.1%)	0.6810
Wraps/tortillas	117 (43.0%)	(57.0%)	108 (41.7%)	151 (58.3%)	0.8266
Rice waffles	8 (2.9%)	(97.1%)	4 (1.5%)	255 (98.5%)	0.4292
Crackers	65 (23.9%)	(76.1%)	63 (24.3%)	196 (75.7%)	0.9092
Chips, crisps, puffs	22 (8.1%)	(91.9%)	16 (6.2%)	243 (93.8%)	0.4930
Puff pastry	123 (45.2%)	(54.8%)	113 (43.6%)	146 (56.4%)	0.7787
Cakes	88 (32.4%)	(67.6%)	79 (30.5%)	180 (69.5%)	0.7143
Biscuits/cookies	67 (24.6%)	(75.4%)	62 (23.9%)	197 (76.1%)	0.9333
Fried baked goods (e.g., donuts)	111 (40.8%)	(59.2%)	107 (41.3%)	152 (58.7%)	0.9748
Breakfast cereals (e.g., cornflakes)	17 (6.3%)	(93.7%)	15 (5.8%)	244 (94.2%)	0.9643
Oatmeal	74 (27.2%)	(72.8%)	56 (21.6%)	203 (78.4%)	0.1630

**Table 10:** Declared problems with availability of specific gluten-free (GF) cereal products in the sub-group of female celiac disease (CD) patients declaring general problems with availability of GF products (*n* = 531), stratified by primary place of purchasing major grocery shopping.

Gluten-Free (GF) Cereal Products	Purchasing in Hypermarkets (*n* = 389)		Purchasing in Other Shops than Hypermarkets (*n* = 142)		*p*
	Declared Problems	No Problems Declared	Declared Problems	No Problems Declared	
Bread	136 (35.0%)	253 (65.0%)	53 (37.3%)	89 (62.7%)	0.6882
Flour (for baking or cooking)	139 (35.7%)	250 (64.3%)	56 (39.4%)	86 (60.6%)	0.4959
Pasta	163 (41.9%)	226 (58.1%)	59 (41.5%)	83 (58.5%)	0.9747
Asian-style noodles	44 (11.3%)	345 (88.7%)	13 (9.2%)	129 (90.8%)	0.5808
Dumplings	197 (50.6%)	192 (49.4%)	78 (54.9%)	64 (45.1%)	0.3812
Wraps/tortillas	161 (41.4%)	228 (58.6%)	64 (45.1%)	78 (54.9%)	0.4371
Rice waffles	9 (2.3%)	380 (97.7%)	3 (2.1%)	139 (97.9%)	0.8475
Crackers	87 (22.4%)	302 (77.6%)	41 (28.9%)	101 (71.1%)	0.1506
Chips, crisps, puffs	30 (7.7%)	359 (92.3%)	8 (5.6%)	134 (94.4%)	0.5271
Puff pastry	161 (41.4%)	228 (58.6%)	75 (52.8%)	67 (47.2%)	0.0246
Cakes	116 (29.8%)	273 (70.2%)	51 (35.9%)	91 (64.1%)	0.2175
Biscuits/cookies	78 (20.1%)	311 (79.9%)	51 (35.9%)	91 (64.1%)	0.0002
Fried baked goods (e.g., donuts)	162 (41.6%)	227 (58.4%)	56 (39.4%)	86 (60.6%)	0.7205
Breakfast cereals (e.g., cornflakes)	15 (3.9%)	374 (96.1%)	17 (12.0%)	125 (88.0%)	0.0011
Oatmeal	88 (22.6%)	301 (77.4%)	42 (29.6%)	100 (70.4%)	0.1245

**Table 11:** Declared problems with availability of specific gluten-free (GF) cereal products in the sub-group of female celiac disease (CD) patients declaring general problem with availability of GF products (*n* = 531), stratified by purchasing GF products online.

Gluten-Free (GF) Cereal Products	GF Products Purchased Online (*n* = 433)		GF Products Never Purchased Online (*n* = 98)		*p*
	Declared Problems	No Problems Declared	Declared Problems	No Problems Declared	
Bread	150 (34.6%)	283 (65.4%)	39 (39.8%)	59 (60.2%)	0.3978
Flour (for baking or cooking)	163 (37.6%)	270 (62.4%)	32 (32.7%)	66 (67.3%)	0.4183
Pasta	181 (41.8%)	252 (58.2%)	41 (41.8%)	57 (58.2%)	0.9165
Asian-style noodles	51 (11.8%)	382 (88.2%)	6 (6.1%)	92 (93.9%)	0.1463
Dumplings	233 (53.8%)	200 (46.2%)	42 (42.9%)	56 (57.1%)	0.0646
Wraps/tortillas	193 (44.6%)	240 (55.4%)	32 (32.7%)	66 (67.3%)	0.0411
Rice waffles	9 (2.1%)	424 (97.9%)	3 (3.1%)	95 (96.9%)	0.8302
Crackers	104 (24.0%)	329 (76.0%)	24 (24.5%)	74 (75.5%)	0.9748
Chips, crisps, puffs	34 (7.9%)	399 (92.1%)	4 (4.1%)	94 (95.9%)	0.2753
Puff pastry	191 (44.1%)	242 (55.9%)	45 (45.9%)	53 (54.1%)	0.8320
Cakes	133 (30.7%)	300 (69.3%)	34 (34.7%)	64 (65.3%)	0.5143
Biscuits/cookies	104 (24.0%)	329 (76.0%)	25 (25.5%)	73 (74.5%)	0.8556
Fried baked goods (e.g., donuts)	185 (42.7%)	248 (57.3%)	33 (33.7%)	65 (66.3%)	0.1257
Breakfast cereals (e.g., cornflakes)	26 (6.0%)	407 (94.0%)	6 (6.1%)	92 (93.9%)	0.8495
Oatmeal	108 (24.9%)	325 (75.1%)	22 (22.4%)	76 (77.6%)	0.6976

**Table 12:** Declared problems with availability of specific gluten-free (GF) cereal products in the sub-group of female celiac disease (CD) patients purchasing specific products, stratified by the frequency of buying them.

Gluten-Free (GF) Cereal Products	Rarely		Sometimes		Often		*p*
	Declared Problems	No Problems Declared	Declared Problems	No Problems Declared	Declared Problems	No Problems Declared	
Bread (*n* = 511)	29 (29.0%)	71 (71.0%)	35 (38.9%)	55 (61.1%)	122 (38.0%)	199 (62.0%)	<0.0001
Flour (for baking or cooking) (*n* = 514)	23 (42.6%)	31 (57.4%)	40 (31.5%)	87 (68.5%)	123 (36.9%)	210 (63.1%)	0.3245
Pasta (*n* = 518)	11 (26.2%)	31 (73.8%)	57 (37.3%)	96 (62.7%)	153 (47.4%)	170 (52.6%)	0.0936
Asian-style noodles (*n* = 278)	8 (7.0%)	106 (93.0%)	11 (12.4%)	78 (87.6%)	16 (21.3%)	59 (78.7%)	0.0148
Dumplings (*n* = 240)	96 (56.8%)	73 (43.2%)	36 (63.2%)	21 (36.8%)	9 (64.3%)	5 (35.7%)	0.6383
Wraps/tortillas (*n* = 294)	84 (44.9%)	103 (55.1%)	43 (47.8%)	47 (52.2%)	14 (82.4%)	3 (17.6%)	0.0126
Rice waffles (*n* = 478)	2 (1.7%)	116 (98.3%)	4 (2.4%)	166 (97.6%)	5 (2.6%)	185 (97.4%)	0.8663
Crackers (*n* = 259)	36 (20.2%)	142 (79.8%)	19 (26.0%)	54 (74.0%)	4 (50.0%)	4 (50.0%)	0.1071
Chips, crisps, puffs (*n* = 462)	7 (5.1%)	131 (94.9%)	18 (9.0%)	183 (91.0%)	11 (8.9%)	112 (91.1%)	0.3631
Puff pastry (*n* = 182)	67 (45.3%)	81 (54.7%)	19 (63.3%)	11 (36.7%)	3 (75.0%)	1 (25.0%)	0.1124
Cakes (*n* = 312)	68 (30.5%)	155 (69.5%)	39 (52.0%)	36 (48.0%)	3 (21.4%)	11 (78.6%)	0.0018
Biscuits/cookies (*n* = 478)	41 (22.7%)	140 (77.3%)	50 (24.5%)	154 (75.5%)	25 (26.9%)	68 (73.1%)	0.7375
Fried baked goods (e.g., donuts) (*n* = 193)	74 (50.7%)	72 (49.3%)	19 (43.2%)	25 (56.8%)	3 (100.0%)	0 (0.0%)	0.1465
Breakfast cereals (e.g., cornflakes) (*n* = 439)	6 (4.4%)	130 (95.6%)	12 (7.3%)	153 (92.7%)	7 (5.1%)	131 (94.9%)	0.5270
Oatmeal (*n* = 397)	23 (22.1%)	81 (77.9%)	32 (26.2%)	90 (73.8%)	53 (31.0%)	118 (69.0%)	0.2646

## Data Availability

Data are provided on request.
